# Supplementary material for: Gender inequalities among authors who contributed equally
Source: eLife. 2019 Jan 30;8:e36399. doi: 10.7554/eLife.36399 (PMC6353592; doi:10.7554/eLife.36399)
Supplement: Figure 2—source data 1. [file elife-36399-fig2-data1.docx]

**Source data Figure 2: Distribution of Bias in papers with two equally contributing authors of different gender by country and year of publication:**

|  |  | | |  |  |  |
| --- | --- | --- | --- | --- | --- | --- |
|  | **No Gender Bias Present** | | **Gender Bias Present** | | **Total** | |
| ***Publication Year*** | No. | % | No. | % | No. | % |
| 1995-2006 | 103 | 35.2 | 190 | ***64.8*** | 293 | 100 |
| 2007+ | 322 | 47.5 | 356 | ***52.5*** | 678 | 100 |
| Total | 425 | 43.8 | 546 | 56.2 | 971 | 100 |
|  |  |  |  |  |  |  |
| ***Country*** | No. | % | No. | % | No. | % |
| USA | 229 | 44 | 292 | **56.0** | 521 | 100 |
| Europe | 144 | 42.2 | 197 | **57.8** | 341 | 100 |
| Other | 52 | 47.7 | 57 | **52.3** | 109 | 100 |
| Total | 425 | 43.8 | 546 | 56.2 | 971 | 100 |

The table above shows that there is suggested decrease in gender bias over time among publications with two equally contributing authors (65% in 1995-2006 vs. 53% in 2007+). However, the frequency of the bias is similar by country of origin.

**Figure 2: Temporal trend in gender bias among two equally contributing authors of different gender (logit of bias (i.e. p(bias)/[1-p(bias)] is plotted on Y-axis against publication year on X-axis)**
