# Supplementary material for: Gender inequalities among authors who contributed equally
Source: eLife. 2019 Jan 30;8:e36399. doi: 10.7554/eLife.36399 (PMC6353592; doi:10.7554/eLife.36399)
Supplement: Figure 2—source data 2. [file elife-36399-fig2-data2.docx]

-----------------------------------------------------------------------------------------

name: <unnamed>

log: /Users/Gayane/Documents/Gayane/My Documents/Consulting/Arturo_Casadevall/Out

> puts/analysis_gender_bias_nov2018.log

log type: text

opened on: 21 Nov 2018, 10:30:59

.

.

. ***************Two-Author Publications************

.

. use pubs_dat2.dta, clear

.

. /*frequency of bias among 2-author publications*/

. tab bias2

Gender Bias |

among 2 |

Authors | Freq. Percent Cum.

------------+-----------------------------------

0 | 424 43.62 43.62

1 | 548 56.38 100.00

------------+-----------------------------------

Total | 972 100.00

.

. /*goodness of fit test*/

. tab bias2

Gender Bias |

among 2 |

Authors | Freq. Percent Cum.

------------+-----------------------------------

0 | 424 43.62 43.62

1 | 548 56.38 100.00

------------+-----------------------------------

Total | 972 100.00

. csgof bias2, expperc(50 50)

+-------------------------------------+

| bias2 expperc expfreq obsfreq |

|-------------------------------------|

| 0 50 486 424 |

| 1 50 486 548 |

+-------------------------------------+

chisq(1) is 15.82, p = .0001

.

.

. /*bias by 2-category year*/

. tab bias2 year2cat, col

+-------------------+

| Key |

|-------------------|

| frequency |

| column percentage |

+-------------------+

Gender |

Bias among | year2cat

2 Authors | 1995-2006 2007+ | Total

-----------+----------------------+----------

0 | 103 321 | 424

| 35.15 47.28 | 43.62

-----------+----------------------+----------

1 | 190 358 | 548

| 64.85 52.72 | 56.38

-----------+----------------------+----------

Total | 293 679 | 972

| 100.00 100.00 | 100.00

.

. bysort year2cat: tab bias2

-----------------------------------------------------------------------------------------

-> year2cat = 1995-2006

Gender Bias |

among 2 |

Authors | Freq. Percent Cum.

------------+-----------------------------------

0 | 103 35.15 35.15

1 | 190 64.85 100.00

------------+-----------------------------------

Total | 293 100.00

-----------------------------------------------------------------------------------------

-> year2cat = 2007+

Gender Bias |

among 2 |

Authors | Freq. Percent Cum.

------------+-----------------------------------

0 | 321 47.28 47.28

1 | 358 52.72 100.00

------------+-----------------------------------

Total | 679 100.00

.

. /*goodness of fit tests by year*/

. preserve

.

. keep if year2cat==0

(2,147 observations deleted)

. csgof bias2 , expperc(50 50) /*1995-2006*/

+-------------------------------------+

| bias2 expperc expfreq obsfreq |

|-------------------------------------|

| 0 50 146.5 103 |

| 1 50 146.5 190 |

+-------------------------------------+

chisq(1) is 25.83, p = 0

.

. restore

.

. preserve

.

. keep if year2cat==1

(830 observations deleted)

. csgof bias2 , expperc(50 50) /*2007-2017*/

+-------------------------------------+

| bias2 expperc expfreq obsfreq |

|-------------------------------------|

| 0 50 339.5 321 |

| 1 50 339.5 358 |

+-------------------------------------+

chisq(1) is 2.02, p = .1556

.

. restore

.

. *to report*

. tabout year2cat bias2 using "Outputs/bias2.rtf", replace c(freq row) ///

> f(0c 1) font(bold)

Table output written to: Outputs/bias2.rtf

Gender Bias among 2 Authors

year2cat 0 0 1 1 Total Total

No. % No. % No. %

1995-2006 103 35.2 190 64.8 293 100.0

2007+ 321 47.3 358 52.7 679 100.0

Total 424 43.6 548 56.4 972 100.0

.

.

. /*bias by 3-category country*/

.

. *to report*

. tabout country3cat bias2 using "Outputs/bias2.rtf", append c(freq row) ///

> f(0c 1) font(bold)

Table output written to: Outputs/bias2.rtf

Gender Bias among 2 Authors

year2cat 0 0 1 1 Total Total

No. % No. % No. %

1995-2006 103 35.2 190 64.8 293 100.0

2007+ 321 47.3 358 52.7 679 100.0

Total 424 43.6 548 56.4 972 100.0

Gender Bias among 2 Authors

country3cat 0 0 1 1 Total Total

No. % No. % No. %

USA 228 43.8 293 56.2 521 100.0

Europe 144 42.1 198 57.9 342 100.0

Other 52 47.7 57 52.3 109 100.0

Total 424 43.6 548 56.4 972 100.0

.

.

. /*Figure 2 - Gender Bias by Year*/

. lowess bias2 year, logit bwidth(0.95) graphregion(color(white)) ytitle("Logit of Bias i

> n First Authorship") ///

> xtitle("Publication Year") lcolor(black) lwidth(thick) title("Extent of Gender Bias in

> First Authorship" "among Two Authors Contributing Equally") ///

> scheme(s2mono)

. graph export "graphs/bias2_year.png", replace

(file graphs/bias2_year.png written in PNG format)

.

. /*GEE models*/

. xtgee bias2 year i.country3cat, i(journal) family(binomial) link(logit) vce(robust) efo

> rm

Iteration 1: tolerance = .00339819

Iteration 2: tolerance = .00009318

Iteration 3: tolerance = 2.543e-06

Iteration 4: tolerance = 6.877e-08

GEE population-averaged model Number of obs = 972

Group variable: journal Number of groups = 19

Link: logit Obs per group:

Family: binomial min = 20

Correlation: exchangeable avg = 51.2

max = 127

Wald chi2(3) = 10.25

Scale parameter: 1 Prob > chi2 = 0.0166

(Std. Err. adjusted for clustering on journal)

------------------------------------------------------------------------------

| Robust

bias2 | Odds Ratio Std. Err. z P>|z| [95% Conf. Interval]

-------------+----------------------------------------------------------------

year | .9583637 .0139078 -2.93 0.003 .931489 .9860138

|

country3cat |

Europe | 1.093036 .2346111 0.41 0.679 .7176803 1.664707

Other | .9173071 .1866618 -0.42 0.671 .6156086 1.366862

|

_cons | 1.64e+37 4.78e+38 2.94 0.003 2.31e+12 1.16e+62

------------------------------------------------------------------------------

Note: _cons estimates baseline odds (conditional on zero random effects).

. est store yrc

.

. xtgee bias2 i.year2cat i.country3cat, i(journal) family(binomial) link(logit) vce(robus

> t) eform

Iteration 1: tolerance = .00085945

Iteration 2: tolerance = .00002644

Iteration 3: tolerance = 8.203e-07

GEE population-averaged model Number of obs = 972

Group variable: journal Number of groups = 19

Link: logit Obs per group:

Family: binomial min = 20

Correlation: exchangeable avg = 51.2

max = 127

Wald chi2(3) = 12.09

Scale parameter: 1 Prob > chi2 = 0.0071

(Std. Err. adjusted for clustering on journal)

------------------------------------------------------------------------------

| Robust

bias2 | Odds Ratio Std. Err. z P>|z| [95% Conf. Interval]

-------------+----------------------------------------------------------------

year2cat |

2007+ | .6052726 .0966877 -3.14 0.002 .4425659 .8277974

|

country3cat |

Europe | 1.098588 .2356274 0.44 0.661 .7215519 1.67264

Other | .904828 .1791936 -0.50 0.614 .6137516 1.33395

|

_cons | 1.804801 .3618777 2.94 0.003 1.218306 2.673637

------------------------------------------------------------------------------

Note: _cons estimates baseline odds (conditional on zero random effects).

. est store yrcat

.

. esttab yrc yrcat using "Outputs/models_bias2.rtf", rtf eform replace ci ///

> drop(_cons) mtitles("Model 1" "Model 2" ) label

(output written to Outputs/models_bias2.rtf)

.

.

. /*Figure 3: predicted marginal probabilities of gender bias among 2-author publication

> s ///

> by year and county*/

. xtgee bias2 c.year i.country3cat, i(journal) family(binomial) link(logit) vce(robust) e

> form

Iteration 1: tolerance = .00339819

Iteration 2: tolerance = .00009318

Iteration 3: tolerance = 2.542e-06

Iteration 4: tolerance = 6.961e-08

GEE population-averaged model Number of obs = 972

Group variable: journal Number of groups = 19

Link: logit Obs per group:

Family: binomial min = 20

Correlation: exchangeable avg = 51.2

max = 127

Wald chi2(3) = 10.25

Scale parameter: 1 Prob > chi2 = 0.0166

(Std. Err. adjusted for clustering on journal)

------------------------------------------------------------------------------

| Robust

bias2 | Odds Ratio Std. Err. z P>|z| [95% Conf. Interval]

-------------+----------------------------------------------------------------

year | .9583637 .0139078 -2.93 0.003 .931489 .9860138

|

country3cat |

Europe | 1.093036 .2346111 0.41 0.679 .7176803 1.664707

Other | .9173071 .1866618 -0.42 0.671 .6156086 1.366862

|

_cons | 1.64e+37 4.78e+38 2.94 0.003 2.31e+12 1.16e+62

------------------------------------------------------------------------------

Note: _cons estimates baseline odds (conditional on zero random effects).

. margins, over(year country3cat)

Predictive margins Number of obs = 972

Model VCE : Robust

Expression : Pr(bias2 != 0), predict()

over : year country3cat

----------------------------------------------------------------------------------

| Delta-method

| Margin Std. Err. z P>|z| [95% Conf. Interval]

-----------------+----------------------------------------------------------------

year#country3cat |

1995#USA | .6997329 .0572713 12.22 0.000 .5874831 .8119826

1995#Europe | .7180855 .0434209 16.54 0.000 .6329821 .803189

1995#Other | .6812915 .0640106 10.64 0.000 .555833 .8067499

1996#USA | .6907223 .055475 12.45 0.000 .5819933 .7994513

1996#Europe | .709397 .0416554 17.03 0.000 .6277539 .79104

1997#USA | .6815643 .0535999 12.72 0.000 .5765104 .7866183

1997#Europe | .7005523 .0398428 17.58 0.000 .6224618 .7786427

1997#Other | .6625452 .0614658 10.78 0.000 .5420744 .7830161

1998#USA | .6722639 .0516563 13.01 0.000 .5710195 .7735083

1998#Europe | .6915554 .0380008 18.20 0.000 .6170753 .7660355

1998#Other | .6529721 .060187 10.85 0.000 .5350078 .7709364

1999#USA | .6628262 .0496562 13.35 0.000 .5655018 .7601506

1999#Europe | .6824108 .0361512 18.88 0.000 .6115558 .7532659

1999#Other | .6432737 .058925 10.92 0.000 .5277829 .7587645

2000#USA | .6532569 .0476145 13.72 0.000 .5599343 .7465795

2000#Europe | .6731232 .0343214 19.61 0.000 .6058545 .740392

2000#Other | .6334563 .0576976 10.98 0.000 .5203711 .7465416

2001#USA | .6435621 .0455486 14.13 0.000 .5542884 .7328357

2001#Europe | .6636979 .0325453 20.39 0.000 .5999104 .7274854

2002#USA | .6337481 .0434797 14.58 0.000 .5485295 .7189668

2002#Europe | .6541405 .0308639 21.19 0.000 .5936483 .7146326

2002#Other | .6134927 .0554245 11.07 0.000 .5048627 .7221226

2003#USA | .623822 .0414326 15.06 0.000 .5426155 .7050284

2003#Europe | .6444569 .0293269 21.97 0.000 .5869771 .7019366

2003#Other | .6033611 .0544198 11.09 0.000 .4967002 .7100219

2004#USA | .6137907 .0394371 15.56 0.000 .5364955 .6910859

2004#Europe | .6346536 .027992 22.67 0.000 .5797903 .6895169

2004#Other | .5931401 .0535312 11.08 0.000 .4882208 .6980593

2005#USA | .6036619 .0375277 16.09 0.000 .5301089 .6772148

2005#Europe | .6247374 .0269227 23.20 0.000 .5719699 .6775049

2005#Other | .5828377 .0527794 11.04 0.000 .4793919 .6862835

2006#USA | .5934434 .0357449 16.60 0.000 .5233847 .663502

2006#Europe | .6147155 .0261841 23.48 0.000 .5633957 .6660353

2006#Other | .5724626 .052184 10.97 0.000 .4701839 .6747413

2007#USA | .5831433 .0341341 17.08 0.000 .5162417 .650045

2007#Europe | .6045953 .0258346 23.40 0.000 .5539605 .6552301

2007#Other | .5620233 .0517622 10.86 0.000 .4605713 .6634753

2008#USA | .5727702 .0327454 17.49 0.000 .5085905 .6369499

2008#Europe | .5943847 .0259168 22.93 0.000 .5435888 .6451806

2008#Other | .5515288 .0515286 10.70 0.000 .4505346 .652523

2009#USA | .5623327 .0316303 17.78 0.000 .5003384 .624327

2009#Europe | .5840918 .0264492 22.08 0.000 .5322523 .6359313

2009#Other | .5409882 .0514939 10.51 0.000 .4400621 .6419143

2010#USA | .5518397 .0308384 17.89 0.000 .4913976 .6122819

2010#Europe | .573725 .0274236 20.92 0.000 .5199757 .6274743

2010#Other | .5304109 .0516642 10.27 0.000 .4291508 .6316709

2011#USA | .5413004 .0304111 17.80 0.000 .4816958 .600905

2011#Europe | .5632931 .028808 19.55 0.000 .5068304 .6197558

2011#Other | .5198061 .0520413 9.99 0.000 .4178071 .6218051

2012#USA | .5307239 .0303759 17.47 0.000 .4711882 .5902597

2012#Europe | .5528048 .0305548 18.09 0.000 .4929185 .6126911

2012#Other | .5091834 .0526215 9.68 0.000 .4060471 .6123197

2013#USA | .5201198 .0307422 16.92 0.000 .4598662 .5803734

2013#Europe | .5422693 .0326092 16.63 0.000 .4783565 .6061821

2013#Other | .4985525 .0533972 9.34 0.000 .393896 .603209

2014#USA | .5094976 .0314993 16.17 0.000 .4477601 .571235

2014#Europe | .5316959 .0349161 15.23 0.000 .4632615 .6001303

2014#Other | .4879228 .0543561 8.98 0.000 .3813868 .5944589

2015#USA | .4988667 .0326191 15.29 0.000 .4349345 .562799

2015#Europe | .5210939 .0374245 13.92 0.000 .4477433 .5944446

2015#Other | .4773041 .0554831 8.60 0.000 .3685593 .5860489

2016#USA | .4882369 .034061 14.33 0.000 .4214786 .5549952

2016#Europe | .5104729 .0400887 12.73 0.000 .4319006 .5890453

2016#Other | .4667058 .0567602 8.22 0.000 .3554579 .5779537

2017#USA | .4776177 .0357776 13.35 0.000 .4074949 .5477405

2017#Europe | .4998425 .0428691 11.66 0.000 .4158206 .5838644

----------------------------------------------------------------------------------

. mplotoffset, offset(0.15) title("") legend(subtitle("Country/Continent") row(1)) graphr

> egion(color(white)) ytitle("Predicted Probability of Gender Bias") ///

> xtitle("publication year") xlab(1995(5)2017) title("Predicted Probability of Gender Bia

> s in First Authorship" "among Two Authors Contributing Equally") ///

> plot1opts(lpattern(solid) mlabel() lcolor(blue) msymbol(circle) lwidth(medthick) mcolor

> (blue) msize(medsmall)) ci1opts(lcolor(blue)) ///

> plot2opts(lpattern(dash) lcolor(green) msymbol(circle_hollow) lwidth(medthick) mcolor(

> green) msize(medsmall)) ci2opts(lcolor(green)) ///

> plot3opts(lpattern(dot) lcolor(red) msymbol(triangle_hollow) lwidth(thick) mcolor(red)

> msize(medsmall)) ci3opts(lcolor(red))

Variables that uniquely identify margins: year country3cat

.

. graph export "graphs/margins_plot_country_year.png", replace

(file graphs/margins_plot_country_year.png written in PNG format)

.

.

. ***************Publications with More than Two Authors***************

.

. use pubs_dat2.dta, clear

.

. tab bias3 F, m

Gender |

Bias among | More than 2 authors

>2 Authors | 0 1 | Total

-----------+----------------------+----------

0 | 0 148 | 148

1 | 0 201 | 201

. | 2,485 143 | 2,628

-----------+----------------------+----------

Total | 2,485 492 | 2,977

.

. tab bias3

Gender Bias |

among >2 |

Authors | Freq. Percent Cum.

------------+-----------------------------------

0 | 148 42.41 42.41

1 | 201 57.59 100.00

------------+-----------------------------------

Total | 349 100.00

.

.

. /*goodness of fit test*/

. tab bias3

Gender Bias |

among >2 |

Authors | Freq. Percent Cum.

------------+-----------------------------------

0 | 148 42.41 42.41

1 | 201 57.59 100.00

------------+-----------------------------------

Total | 349 100.00

. csgof bias3, expperc(50 50)

+-------------------------------------+

| bias3 expperc expfreq obsfreq |

|-------------------------------------|

| 0 50 174.5 148 |

| 1 50 174.5 201 |

+-------------------------------------+

chisq(1) is 8.05, p = .0046

.

. tabout year2cat bias3 using "Outputs/bias3.rtf", replace c(freq row) ///

> f(0c 1) font(bold)

Table output written to: Outputs/bias3.rtf

Gender Bias among >2 Authors

year2cat 0 0 1 1 Total Total

No. % No. % No. %

1995-2006 22 37.3 37 62.7 59 100.0

2007+ 126 43.4 164 56.6 290 100.0

Total 148 42.4 201 57.6 349 100.0

. tabout country3cat bias3 using "Outputs/bias3.rtf", append c(freq row) ///

> f(0c 1) font(bold)

Table output written to: Outputs/bias3.rtf

Gender Bias among >2 Authors

year2cat 0 0 1 1 Total Total

No. % No. % No. %

1995-2006 22 37.3 37 62.7 59 100.0

2007+ 126 43.4 164 56.6 290 100.0

Total 148 42.4 201 57.6 349 100.0

Gender Bias among >2 Authors

country3cat 0 0 1 1 Total Total

No. % No. % No. %

USA 67 39.9 101 60.1 168 100.0

Europe 60 51.3 57 48.7 117 100.0

Other 21 32.8 43 67.2 64 100.0

Total 148 42.4 201 57.6 349 100.0

. /*Open with Excel to copy in word*/

.

. lowess bias3 year, logit bwidth(0.98) graphregion(color(white)) ytitle("Logit of Bias i

> n First Authorship") ///

> xtitle("Publication Year") lcolor(black) lwidth(thick) title("Extent of Gender Bias in

> First Authorship" "among More than Two Authors Contributing Equally") ///

> scheme(s2mono)

. graph export "graphs/bias3_year.png", replace

(file graphs/bias3_year.png written in PNG format)

.

. /*GEE models*/

. xtgee bias3 year i.country3cat, i(journal) family(binomial) link(logit) vce(robust) efo

> rm

Iteration 1: tolerance = .51684429

Iteration 2: tolerance = .07129471

Iteration 3: tolerance = .00391765

Iteration 4: tolerance = .00016212

Iteration 5: tolerance = 6.121e-06

Iteration 6: tolerance = 2.248e-07

GEE population-averaged model Number of obs = 349

Group variable: journal Number of groups = 19

Link: logit Obs per group:

Family: binomial min = 5

Correlation: exchangeable avg = 18.4

max = 55

Wald chi2(3) = 6.14

Scale parameter: 1 Prob > chi2 = 0.1050

(Std. Err. adjusted for clustering on journal)

------------------------------------------------------------------------------

| Robust

bias3 | Odds Ratio Std. Err. z P>|z| [95% Conf. Interval]

-------------+----------------------------------------------------------------

year | .9900123 .0300151 -0.33 0.741 .9328975 1.050624

|

country3cat |

Europe | .6596108 .1683905 -1.63 0.103 .3999323 1.0879

Other | 1.410454 .3944625 1.23 0.219 .8152724 2.440141

|

_cons | 9.09e+08 5.54e+10 0.34 0.735 1.14e-43 7.27e+60

------------------------------------------------------------------------------

Note: _cons estimates baseline odds (conditional on zero random effects).

. est store yrc2

.

. xtgee bias3 i.year2cat i.country3cat, i(journal) family(binomial) link(logit) vce(robus

> t) eform

Iteration 1: tolerance = .08313764

Iteration 2: tolerance = .00437613

Iteration 3: tolerance = .00017291

Iteration 4: tolerance = 5.717e-06

Iteration 5: tolerance = 1.804e-07

GEE population-averaged model Number of obs = 349

Group variable: journal Number of groups = 19

Link: logit Obs per group:

Family: binomial min = 5

Correlation: exchangeable avg = 18.4

max = 55

Wald chi2(3) = 6.25

Scale parameter: 1 Prob > chi2 = 0.1002

(Std. Err. adjusted for clustering on journal)

------------------------------------------------------------------------------

| Robust

bias3 | Odds Ratio Std. Err. z P>|z| [95% Conf. Interval]

-------------+----------------------------------------------------------------

year2cat |

2007+ | .8369502 .2523597 -0.59 0.555 .4634926 1.51132

|

country3cat |

Europe | .6559698 .1702098 -1.62 0.104 .3944716 1.090817

Other | 1.399826 .3877519 1.21 0.225 .8133757 2.409112

|

_cons | 1.811936 .6417077 1.68 0.093 .905076 3.627445

------------------------------------------------------------------------------

Note: _cons estimates baseline odds (conditional on zero random effects).

. est store yrcat2

.

. esttab yrc2 yrcat2 using "Outputs/models_bias3.rtf", rtf eform replace ci ///

> drop(_cons) mtitles("Model 1" "Model 2" ) label

(output written to Outputs/models_bias3.rtf)

.

.

.

. *************ALPHABETICAL ORDER****************

.

. /*frequency of alphabetical order*/

. tab alphabetical

alphabetica |

l | Freq. Percent Cum.

------------+-----------------------------------

0 | 1,629 54.72 54.72

1 | 1,348 45.28 100.00

------------+-----------------------------------

Total | 2,977 100.00

.

.

. /*2-author publications*/

. tab mm alphabetical, row

+----------------+

| Key |

|----------------|

| frequency |

| row percentage |

+----------------+

| alphabetical

mm | 0 1 | Total

-----------+----------------------+----------

0 | 1,114 863 | 1,977

| 56.35 43.65 | 100.00

-----------+----------------------+----------

1 | 515 485 | 1,000

| 51.50 48.50 | 100.00

-----------+----------------------+----------

Total | 1,629 1,348 | 2,977

| 54.72 45.28 | 100.00

. tab mf alphabetical, row

+----------------+

| Key |

|----------------|

| frequency |

| row percentage |

+----------------+

| alphabetical

mf | 0 1 | Total

-----------+----------------------+----------

0 | 1,350 1,079 | 2,429

| 55.58 44.42 | 100.00

-----------+----------------------+----------

1 | 279 269 | 548

| 50.91 49.09 | 100.00

-----------+----------------------+----------

Total | 1,629 1,348 | 2,977

| 54.72 45.28 | 100.00

. tab fm alphabetical, row

+----------------+

| Key |

|----------------|

| frequency |

| row percentage |

+----------------+

| alphabetical

fm | 0 1 | Total

-----------+----------------------+----------

0 | 1,409 1,144 | 2,553

| 55.19 44.81 | 100.00

-----------+----------------------+----------

1 | 220 204 | 424

| 51.89 48.11 | 100.00

-----------+----------------------+----------

Total | 1,629 1,348 | 2,977

| 54.72 45.28 | 100.00

. tab ff alphabetical, row

+----------------+

| Key |

|----------------|

| frequency |

| row percentage |

+----------------+

| alphabetical

ff | 0 1 | Total

-----------+----------------------+----------

0 | 1,460 1,140 | 2,600

| 56.15 43.85 | 100.00

-----------+----------------------+----------

1 | 169 208 | 377

| 44.83 55.17 | 100.00

-----------+----------------------+----------

Total | 1,629 1,348 | 2,977

| 54.72 45.28 | 100.00

.

.

. /*more than 2-author publications*/

. tab au3male alphabetical, row

+----------------+

| Key |

|----------------|

| frequency |

| row percentage |

+----------------+

All male |

authors |

among >2 | alphabetical

authors | 0 1 | Total

-----------+----------------------+----------

1 | 91 25 | 116

| 78.45 21.55 | 100.00

-----------+----------------------+----------

Total | 91 25 | 116

| 78.45 21.55 | 100.00

. tab au3female alphabetical, row

+----------------+

| Key |

|----------------|

| frequency |

| row percentage |

+----------------+

All female |

authors |

among >2 | alphabetical

authors | 0 1 | Total

-----------+----------------------+----------

1 | 16 11 | 27

| 59.26 40.74 | 100.00

-----------+----------------------+----------

Total | 16 11 | 27

| 59.26 40.74 | 100.00

. tab m alphabetical, row

+----------------+

| Key |

|----------------|

| frequency |

| row percentage |

+----------------+

First |

position |

male among | alphabetical

>2 authors | 0 1 | Total

-----------+----------------------+----------

1 | 148 53 | 201

| 73.63 26.37 | 100.00

-----------+----------------------+----------

Total | 148 53 | 201

| 73.63 26.37 | 100.00

. tab f alphabetical, row

+----------------+

| Key |

|----------------|

| frequency |

| row percentage |

+----------------+

First |

position |

female |

among >2 | alphabetical

authors | 0 1 | Total

-----------+----------------------+----------

1 | 111 37 | 148

| 75.00 25.00 | 100.00

-----------+----------------------+----------

Total | 111 37 | 148

| 75.00 25.00 | 100.00

.

.

. log close

name: <unnamed>

log: /Users/Gayane/Documents/Gayane/My Documents/Consulting/Arturo_Casadevall/Out

> puts/analysis_gender_bias_nov2018.log

log type: text

closed on: 21 Nov 2018, 10:31:07

-----------------------------------------------------------------------------------------
