# Supplementary material for: Gender inequalities among authors who contributed equally
Source: eLife. 2019 Jan 30;8:e36399. doi: 10.7554/eLife.36399 (PMC6353592; doi:10.7554/eLife.36399)
Supplement: Figure 2—source data 3. [file elife-36399-fig2-data3.docx]

-----------------------------------------------------------------------------------------

name: <unnamed>

log: /Users/Gayane/Documents/Gayane/My Documents/Consulting/Arturo_Casadevall/Out

> puts/data_management_gender_bias_nov2018.log

log type: text

opened on: 21 Nov 2018, 10:30:54

.

. import excel "Data set final Casadevall Broderick.xlsx", sheet("Sheet1") firstrow clear

.

. drop if PaperID=="Totals"

(1 observation deleted)

.

.

. *************data manipulation/new variables for analysis***************

. label drop _all

.

. gen country3cat = 2

. replace country3cat = 1 if regexm(country, "(Austria|Belgium|Croatia|Czech|Demmark|Denm

> ark|Finland|France|Framce|Germany|Greece|Ireland|Italy|Hungary|Iceland|Luxemburg|Nether

> lands|Nethelands|Poland|Portugal|Spain|Sweden|Sweeden|Switzerland|Swi|Netherlands|Norwa

> y|Russia|Slovenia|UK|United Kingdom)")

(1,020 real changes made)

. replace country3cat = 0 if regexm(country, "USA") | regexm(country, "UCLA")

(1,522 real changes made)

.

. label define cntry 1 "Europe" 2 "Other" 0 "USA"

. label values country3cat cntry

.

. /*to check

> bysort country3cat: tab country

> */

.

. gen author2 = 0

. replace author2 = 1 if mf==1 | fm==1 | mm==1 | ff==1

(2,349 real changes made)

. label var author2 "Two equally contributing authors"

.

. gen bias2 = 0 if mf==1 | fm==1

(2,005 missing values generated)

. replace bias2 = 1 if mf==1

(548 real changes made)

. label var bias2 "Gender Bias among 2 Authors"

.

.

. rename PaperID id

.

. /*journal*/

. gen journal = 1 if regexm(id, "Biophys")

(2,876 missing values generated)

. replace journal = 2 if regexm(id, "CellReport")

(105 real changes made)

. replace journal = 3 if regexm(id, "CurrentBio")

(103 real changes made)

. replace journal = 4 if regexm(id, "JBC") /*Journal of Biol Chemistry"*/

(300 real changes made)

. replace journal = 5 if regexm(id, "JCB") /*Journal of Cell Biology"*/

(101 real changes made)

. replace journal = 6 if regexm(id, "JCI") /*Journal of Clin Investigation*/

(121 real changes made)

. replace journal = 7 if regexm(id, "JEM") /*Journal of Exp Med*/

(210 real changes made)

. replace journal = 8 if regexm(id, "JI") /*Journal of Immunol*/

(308 real changes made)

. replace journal = 9 if regexm(id, "Misc") /*MISC*/

(120 real changes made)

. replace journal = 10 if regexm(id, "Nature") /*Nature*/

(104 real changes made)

. replace journal = 11 if regexm(id, "PLOSBio") /*PLOS Bio*/

(110 real changes made)

. replace journal = 12 if regexm(id, "PLOSCompBio")

(95 real changes made)

. replace journal = 13 if regexm(id, "PLOSGenetics")

(186 real changes made)

. replace journal = 14 if regexm(id, "PLOSNTD") /*PLOS Negl Top Dis*/

(105 real changes made)

. replace journal = 15 if regexm(id, "PLOSPath") /*PLOS Pathogen*/

(179 real changes made)

. replace journal = 16 if regexm(id, "PNAS")

(411 real changes made)

. replace journal = 17 if regexm(id, "Science")

(128 real changes made)

. replace journal = 18 if regexm(id, "eLife")

(90 real changes made)

. replace journal = 19 if regexm(id, "mBio")

(100 real changes made)

.

. label define jr ///

> 1 "Biophysical J" ///

> 2 "Cell Reports" ///

> 3 "Current Biology" ///

> 4 "Journal of Biol Chemistry" ///

> 5 "Journal of Cell Biology" ///

> 6 "Journal of Clin Investigation" ///

> 7 "Journal of Exp Med" ///

> 8 "Journal of Immunol" ///

> 9 "MISC" ///

> 10 "Nature" ///

> 11 "PLOS Bio" ///

> 12 "PLOS Comp Bio" ///

> 13 "PLOS Genetics" ///

> 14 "PLOS Negl Top Dis" ///

> 15 "PLOS Pathogen" ///

> 16 "PNAS" ///

> 17 "Science" ///

> 18 "eLife" ///

> 19 "mBio"

.

. label values journal jr

.

. /*to check

> bysort journal: tab id

> */

.

. /*year of publication*/

. replace year = 2013 if year==2103 /*looked up the paper*/

(1 real change made)

.

. gen year2cat = year>=2007

. replace year2cat = . if year==.

(0 real changes made)

. label define yr 0 "1995-2006" 1 "2007+"

. label values year2cat yr

. label var year "Publication Year"

.

.

. label var F "More than 2 authors"

.

. label var m "First position male among >2 authors"

. label var f "First position female among >2 authors"

.

. gen bias3 = 0 if F==1 & f==1

(2,829 missing values generated)

. replace bias3 = 1 if F==1 & m==1

(201 real changes made)

. label var bias3 "Gender Bias among >2 Authors"

.

. gen anybias = .

(2,977 missing values generated)

. replace anybias = 1 if bias2==1 | bias3==1

(749 real changes made)

. replace anybias = 0 if (bias2==0 | bias3==0) & anybias==.

(572 real changes made)

.

. gen au3male = 1 if F==1 & m==. & f==. & regexm(Orderfor2au, "m")

(2,861 missing values generated)

. gen au3female = 1 if F==1 & m==. & f==. & regexm(Orderfor2au, "f")

(2,950 missing values generated)

. label var au3male "All male authors among >2 authors"

. label var au3female "All female authors among >2 authors"

.

. /*to check

> tab Orderfor2au if au3male==1

> tab Orderfor2au if au3female==1

> */

.

.

. save pubs_dat2.dta, replace

file pubs_dat2.dta saved

.

. log close

name: <unnamed>

log: /Users/Gayane/Documents/Gayane/My Documents/Consulting/Arturo_Casadevall/Out

> puts/data_management_gender_bias_nov2018.log

log type: text

closed on: 21 Nov 2018, 10:30:54

-----------------------------------------------------------------------------------------
