# Supplementary material for: Gender inequalities among authors who contributed equally
Source: eLife. 2019 Jan 30;8:e36399. doi: 10.7554/eLife.36399 (PMC6353592; doi:10.7554/eLife.36399)
Supplement: Figure 4—source data 1. [file elife-36399-fig4-data1.docx]

**Source data Figure 4: Results of Generalized Linear Population-Average model with Binomial Distribution and Logit-link estimated with Generalized Estimating Equations (GEE) and robust variance estimate with gender bias in first authorship as the dependent variable** **among papers with two equally contributing authors of different gender (N = 971)**

| **Predictors** | **Odds Ratio of Gender Bias in First Authorship**  **(95% Confidence Interval)** | | |
| --- | --- | --- | --- |
|  | Model 1  (year as a continuous variable) | Model 2  (year as a categorical variable) |  |
| **Publication Year** (for every additional year) | 0.958^**^ |  |  |
|  | [0.931,0.986] |  |  |
|  |  |  |  |
| **Publication year**  2007+ vs 1995-2006 |  | 0.600^**^ |  |
|  |  | [0.438,0.821] |  |
|  |  |  |  |
| **Country of Origin** |  |  |  |
| Europe vs US | 1.098 | 1.103 |  |
|  | [0.723,1.668] | [0.727,1.675] |  |
|  |  |  |  |
| Other vs. US | 0.926 | 0.914 |  |
|  | [0.620,1.382] | [0.619,1.349] |  |
|  |  |  |  |
| *N* | 971 | 971 |  |

Exponentiated coefficients; 95% confidence intervals in brackets

^*^ *p* < 0.05, ^**^ *p* < 0.01, ^***^ *p* < 0.001

These results show that among publications with two equally contributing authors of different gender (N = 971), after adjustment for country and accounting for multiple publications by the same journal, for every additional year, the odds of gender bias decreases by estimated 4% (95%CI: from 1 to 7% decrease, p-value = 0.003).

In addition, when looking at 2 categories of publication year, after adjustment for country and accounting for multiple publications by the same journal, the odds of gender bias is estimated to be 40% lower in years 2007+ compared to 1995-2006 (95%CI: from 18% to 56% lower, p-value =0.001).
